# Empirical Evidence for Synchrony in the Evolution of TB Cases and HIV+ Contacts among the San Francisco Homeless

**DOI:** 10.1371/journal.pone.0008851

**Published:** 2010-01-22

**Authors:** Mojdeh Mohtashemi, L. Masae Kawamura

**Affiliations:** 1 MITRE Corporation, McLean, Virginia, United States of America; 2 MIT Computer Science and AI Laboratory, Cambridge, Massachusetts, United States of America; 3 Tuberculosis Control Section, San Francisco Department of Public Health, San Francisco, California, United States of America; National AIDS Research Institute, India

## Abstract

The re-emergence of tuberculosis (TB) in the mid-1980s in many parts of the world, including the United States, is often attributed to the emergence and rapid spread of human immunodeficiency virus (HIV) and acquired immunodeficiency syndrome (AIDS). Although it is well established that TB transmission is particularly amplified in populations with high HIV prevalence, the epidemiology of interaction between TB and HIV is not well understood. This is partly due to the scarcity of HIV-related data, a consequence of the voluntary nature of HIV status reporting and testing, and partly due to current practices of screening high risk populations through separate surveillance programs for HIV and TB. The San Francisco Department of Public Health, TB Control Program, has been conducting active surveillance among the San Francisco high-risk populations since the early 1990s. We present extensive TB surveillance data on HIV and TB infection among the San Francisco homeless to investigate the association between the TB cases and their HIV+ contacts. We applied wavelet coherence and phase analyses to the TB surveillance data from January 1993 through December 2005, to establish and quantify statistical association and synchrony in the highly non-stationary and ostensibly non-periodic waves of TB cases and their HIV+ contacts in San Francisco. When stratified by homelessness, we found that the evolution of TB cases and their HIV+ contacts is highly coherent over time and locked in phase at a specific periodic scale among the San Francisco homeless, but no significant association was observed for the non-homeless. This study confirms the hypothesis that the dynamics of HIV and TB are significantly intertwined and that HIV is likely a key factor in the sustenance of TB transmission among the San Francisco homeless. The findings of this study underscore the importance of contact tracing in detection of HIV+ individuals that may otherwise remain undetected, and thus highlights the ever-increasing need for HIV-related data and an integrative approach to monitoring high-risk populations with respect to HIV and TB transmission.

## Introduction

**Figure 1:**
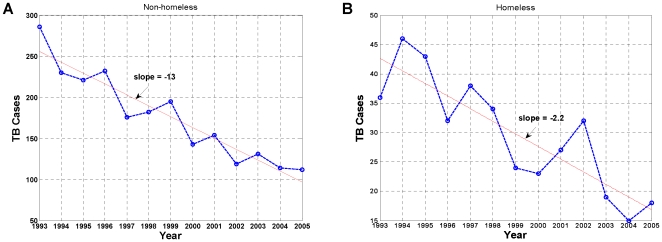
Annual number of TB cases in San Francisco, January 1993–December 2005. (A) Non-homeless: general population, exclusive of homeless. (B) Homeless. The rate of decline over time in the number of TB cases is much faster for the non-homeless than that of the homeless. Red line: linear fit.

San Francisco has the highest rate of TB in the United States, nearly three to four times the national rate [1]. While North America is among the lowest risk regions of the world for TB transmission [2], [3], in some areas of San Francisco, the TB rate is as high as the developing world, with rates ranging from 100 to 200 per 100,000 [1]. After decades of decline in the TB incidence due to advances in health and medicine in the mid-20^th^ century, San Francisco, as many parts of the world, witnessed a gradual re-emergence of TB in the mid-1980s. This re-emergence has been attributed to a number of risk factors, including increased homelessness and prevalence of HIV and AIDS [4]. In the early 1990s, the San Francisco Department of Public Health (SFDPH), TB Control Program (TBCP), adopted new and intensified control measures to significantly reduce the transmission of *Mycobacterium tuberculosis* in San Francisco [5]. While the number of TB cases has been steadily declining since the early 1990s in San Francisco's general population exclusive of the homeless (non-homeless) (Figure 1A), the rate of decline has been much slower and more variable for the homeless population, in spite of extensive active surveillance among the homeless by the SFDPH TBCP (Figure 1B). Although this disparity in trends has been mostly attributed to the prevalence of HIV and AIDS among the indigent population in San Francisco [6], [7], and in many impoverished regions of the world [8]–[17], few studies have been properly designed and documented to establish a direct epidemiological link between TB and HIV.

In a nationwide study, exclusive of California, the Centers for Disease Control and Prevention (CDC) reported that 9% of TB patients were also HIV+ in 2005 [18]. For the San Francisco general population, a recent study using molecular genotyping estimated that 13.7% of TB cases from 1991 to 2002 were attributed to HIV infection [19]. For the San Francisco homeless, in general, and the TB-infected homeless in particular, studies pertaining to the prevalence of HIV are scarce. In a cross-sectional study of the San Francisco inner city shelters and free meal programs, the HIV seroprevalence was estimated at 8.5% (95% Confidence Interval (CI): 7–10%) among the homeless [6]. The authors found that 19% of the study subjects who were seropositive for HIV were co-infected with TB. In a recent study among the San Francisco urban homeless and marginally housed, the HIV seroprevalence was estimated to be five times higher among indigent young adults than in the San Francisco general population [7]. The overall HIV seroprevalence was estimated at 10.5% and 29.6% among men who had sex with men. To date, we are not aware of any documented study on the HIV prevalence, or seroprevalence, among the TB-infected homeless population.

The objective of this study is three-fold. First, using extensive TB surveillance data in San Francisco, we demonstrate that the prevalence of HIV infection is likely higher for the TB-infected homeless population than what is reported for the general population of the United States, the San Francisco general population, and the San Francisco homeless. Second, using wavelet time series analysis of the homeless TB cases and their HIV+ contacts, we show that the dynamics of the two infectious diseases are significantly intertwined and synchronized at a specific periodic band, implying the importance of contact tracing in detection of unknown HIV+ contacts, and a likely indicator of epidemiological interaction between TB and HIV among the San Francisco homeless. Third, we argue that these findings have direct implications for HIV and TB surveillance; they highlight the importance of HIV-related data and call for an integrative approach to the TB and HIV surveillance in high-risk populations.

## Materials and Methods

### Data

The dataset for this study consists of comprehensive information on individuals that have been diagnosed by the SFDPH TBCP with active TB (TB cases), and their reported contacts (PPD+ and PPD−), all identified from January 1993 to December 2005. PPD+ contacts refer to individuals with positive purified protein derivative (PPD) skin test for TB, indicating exposure to the *Mycobacterium tuberculosis*. Likewise, PPD− contacts are individuals with a negative TB skin test result. For the homeless population, there were a total of 387 TB cases and 613 reported contacts in the data. For the non-homeless, there was a total of 2,329 TB cases and 2,003 reported contacts. The TBCP routinely collects comprehensive information on TB cases, including demographics (e.g., age, gender, race), individuals they have been in contact or exposed to (contacts), duration of exposure to each reported contact, personal risk factors (e.g., intravenous drug use, HIV status, alcohol intake), laboratory results (e.g., PPD skin test, chest x-ray), date of diagnosis, and primary residence. Contact tracing is a key component of active and ongoing surveillance by the SFDPH TBCP. TB patients are brought to the TB clinic as they are identified. Contact tracing follows immediately the identification and diagnosis of a TB case. Reported contacts of each TB patient are then tracked and brought to the TB clinic for medical and laboratory examinations and interviews. During these interviews, the SFDPH TBCP documents wide-ranging information, including personal, demographic, risk factors (e.g., HIV status), and exposure-related information. The SFDPH TBCP providers determine the HIV status of the TB cases and their contacts from their medical records. If the HIV status cannot be determined, the SFDPH TBCP will offer HIV testing. Approval was obtained from the SFDPH TBCP to use its routinely collected TB surveillance data before any analysis was performed.

To examine the association between TB and HIV infection among the San Francisco homeless, contact network data comprised of monthly number of TB cases and their reported contacts who are HIV+ but PPD− (HIV+/PPD−) were retrieved for each population. Because we seek to determine the significance of interdependency in the dynamics of HIV and TB infection, PPD+ contacts were excluded from the analysis to reduce bias in statistical correlation.

### Wavelet Coherence and Phase Analysis

We used wavelet time series analysis [20]–[24] to investigate the relationship between the evolution of TB cases and their reported HIV+/PPD− contacts. In this study, each time series undergoing wavelet transform was logarithmically transformed after adding a constant of one to each value in the series. The log-transformed series were then standardized to have zero mean and unit variance before wavelet analysis was performed. To determine the statistical association or correlation between the spectra of a pair of time series [24], we performed wavelet coherence analysis. The resulting coherence values can identify regions of high correlation between a pair of time series at various periodic components. The phase difference between the two time series was then computed to quantify the statistical tendency for the two signals to be phase locked, and to determine the temporal lag between the two [25], [26], if any. Statistical significance of the results was assessed using bootstrap methods [27]. For a complete mathematical treatment of the wavelet coherence and phase analysis, see Appendix S1.

Wavelet coherence and phase analysis have proven to be effective analytic tools for studying the association and synchrony in the evolution of time series data with periodic components. In particular, wavelet phase analysis has been used to study the spatio-temporal patterns of movement of measles epidemics from large cities to smaller towns in the pre- and post-vaccination era in England and Wales [28], and the relationship and spatial synchrony between the dengue incidence in Bangkok and the rest of Thailand [29]. It has also been used to analyze weekly state-specific excess mortality rates from pneumonia and influenza (P&I) from the lower 49 contiguous states in the United States over the past 30 years [30]; the association and synchrony between cholera incidence, Indian Oscillation Index (IOI), and rainfall across five West African countries [31]; and the association and synchrony between the Influenza-Like Illnesses (ILI) consultation rates and laboratory surveillance data [32].

A major theme common in aforementioned investigations is that their data, while non-stationary, comprise strong periodic components, including those generated by the effect of seasonality. In contrast, the TB and HIV time series data documented by the SFDPH TBCP are highly non-stationary and do not comprise any apparent periodic component.

## Results

### Trends in HIV Reporting

While the prevalence, or seroprevalence, of HIV infection among the TB-infected homeless population in San Francisco remains unknown, it is widely believed to be higher than that of the general population of the United States, exclusive of California [18], that of the San Francisco general population [19], or even that of the San Francisco homeless [6], [7]. Indeed, a count of the TB cases co-infected with HIV, derived from the SFDPH TBCP data, strongly supports this hypothesis and reveals contrasting trends between the homeless and non-homeless populations of San Francisco. In the SFDPH TBCP data spanning the 13-year period from January 1993 to December 2005, 37% of the homeless TB cases are also HIV+ (Table 1). The actual rate, however, is likely higher, since 17% of the TB cases in the data have unknown HIV status (Table 1). During the same period, only 10% of the non-homeless TB cases were reported as HIV+, while 54% of them had missing HIV status (Table 1). In 2005, on average, about 10% of all TB cases (homeless and non-homeless) in the SFDPH TBCP data were reported as HIV+ 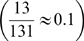 (see last row in Table 1), which is much closer to the average value for the nation, exclusive of California, as reported by the CDC for that year [18]. When stratified by homelessness, however, 44% of the homeless TB cases are reported as HIV+ versus 4% for the non-homeless, an eleven-fold difference (see last row in Table 1), suggesting that the average value is a misleading measure of disease frequency.

**Table 1:** Number of TB cases, HIV+ TB cases, and TB cases with missing HIV status, in the San Francisco homeless and non-homeless populations.

	Homeless			Non-homeless		
	TB cases	HIV+	Unknown HIV Status	TB cases	HIV+	Unknown HIV Status
**1993–2005**	387	144 (37%)	69 (17%)	2329	233 (10%)	1269 (54%)
**1996–2005**	262	95 (36%)	42 (16%)	1579	122 (7.7%)	859 (54%)
**1999–2005**	158	68 (43%)	20 (12%)	982	63 (6.4%)	548 (55.8%)
**2002–2005**	84	36 (43%)	8 (9.5%)	478	33 (7%)	250 (53%)
**2005**	18	8 (44%)	1 (5%)	113	5 (4%)	54 (48%)

Over time, HIV status reporting has improved among the homeless, while it has remained at low levels, and almost constant, among the non-homeless (third column under each population category). At the same time, the percentage of HIV+ TB cases has increased among the homeless, but it has slightly declined among the non-homeless (second column under each population category). To compare against the CDC estimates for 2005 (18), related numbers from the SFDPH TBCP data are also provided for that year.

**Figure 2:**
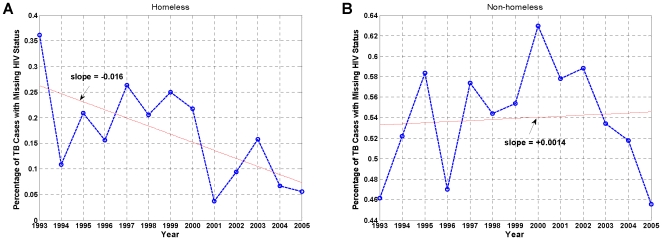
Annual percentage of TB cases with missing HIV status, January 1993–December 2005. (A) Homeless. (B) Non-homeless. While the reporting of HIV status has improved over time among the homeless TB cases (negative slope), it has remained more or less constant among the non-homeless TB cases (small positive slope). Red line: linear fit.

At the same time, the SFDPH TBCP data reveals a disparity in trends of the HIV status reporting between the homeless and non-homeless. Because testing or reporting of HIV status is not mandatory, missing information pertaining to the HIV status of TB cases and their contacts is a limiting factor for investigating the impact of HIV on TB or the association between them. According to the CDC, HIV-status reporting among TB patients has improved in the United States general population exclusive of California [18]. However, when the SFDPH TBCP data are stratified according to each subpopulation, contrasting dynamics are observed. While HIV-status reporting has improved among the homeless TB cases (Figure 2A and Table 1), it has remained relatively constant and at low levels (<50%) among the non-homeless (Figure 2B and Table 1). At the same time, the proportion of homeless TB cases reported as HIV+ has increased either due to improved reporting, rise in the prevalence of HIV infection, or both (Figure S1A and Table 1), but declined for the non-homeless (Figure S1B and Table 1).

**Figure 3:**
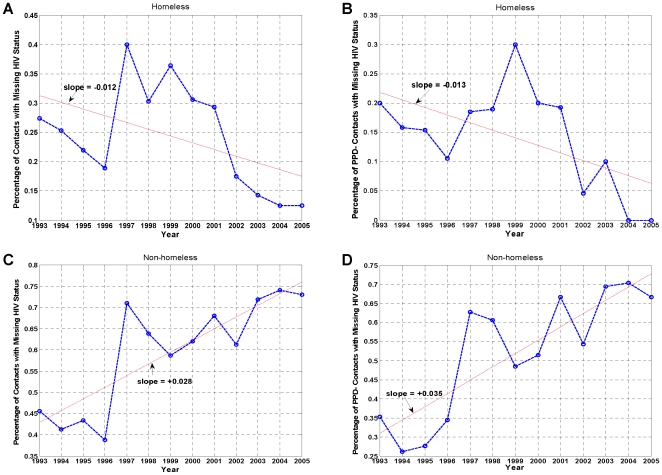
Annual percentage of all contacts and PPD− contacts with missing HIV status, January 1993–December 2005. (A and B) Homeless. (C and D) Non-homeless. In A and C, contacts include both PPD+ and PPD− individuals. While the reporting of HIV status has improved over time among all contacts and PPD− contacts in the homeless population (negative slopes), it has worsened among the non-homeless (positive slopes). Red line: linear fit.

A similar trend is observed for the reported contacts of the TB cases. While HIV-status reporting has improved among reported homeless contacts (Figure 3A), it has worsened among the non-homeless (Figure 3C). The same is true when contacts are stratified according to the PPD skin test results. HIV-status reporting has generally improved for the PPD− homeless contacts, particularly after 1999 (Figure 3B), but the converse is observed for the PPD− non-homeless contacts (Figure 3D). Similar trends are observed for PPD+ contacts in the homeless and non-homeless populations (Figure S2). While it is unclear why the two populations manifest such contrasting behavior, these findings, along with those in Figure 1, suggest that HIV is likely a complicating factor in controlling TB transmission among the homeless, and that HIV-related data for the homeless have come to more reliably capture the underlying population risk behavior and disease frequency among the homeless than that for the non-homeless.

### Association and Synchrony between HIV and TB

**Figure 4:**
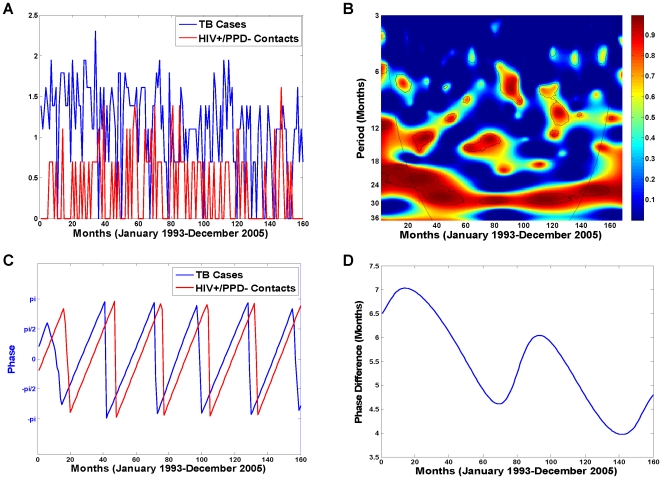
Wavelet time series analysis of the homeless TB cases and their HIV+/PPD− contacts, January 1993–December 2005. Both time series were log-transformed and standardized (mean centered with unit variance) before wavelet analyses were performed. (A) Log-transformed time series of the TB cases and their HIV+/PPD−contacts. (B) Wavelet coherence between the homeless TB cases and their HIV+/PPD−contacts. One periodic component at 27–31 months is present at 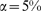 significant level (black lines), computed based on 400 bootstrapped series. The colors code for power values from dark blue, representing low values, to dark red, representing high values; the superimposed parabola is the cone of influence, which measures the extent of edge effects. (C) Wavelet phase evolution of homeless TB cases and their HIV+/PPD− contacts computed at the at the 27–31 months periodic band in radians. (D) Wavelet phase difference of the phases of TB cases and their HIV+/PPD− contacts computed at the 27–31 months periodic band. Waves of TB cases and their HIV+/PPD− contacts are separated, with a mean lag time of 5.4 months over the entire 13-year period, and with a mean lag time of 4.4 months over the four-year period from January 2002 to December 2005, when the HIV related data is most complete.

While the existence of a periodic component is not visibly detectable from the time series plots of the TB cases and their HIV+/PPD− contacts (Figure 4A), wavelet coherence analysis of the two reveals otherwise. Figure 4B is the wavelet coherence plot of the TB cases and their HIV+/PPD− contacts over the 13-year period in the data examined at various periodic scales (3–36 months on the y axis). The coherence between the two time series appears to be strongest at the 27–31 months periodic band and almost consistently significant over the entire 13-year period (see patches of significant signals indicated by black lines along the stretch of red band). This suggests that the two time series are synchronized and locked in phase approximately every 27–31 months.

**Figure 5:**
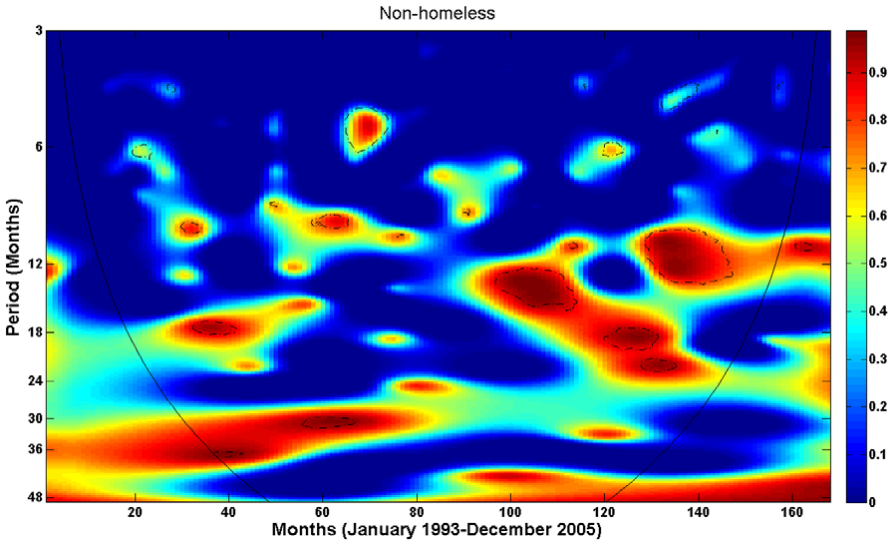
Wavelet coherence between the non-homeless TB cases and their HIV+/PPD− contacts, January 1993–December 2005. Except for patches of high coherence at various periodic scales appearing transiently in time, there is no evidence of consistently significant coherence and synchrony.

To quantify the synchrony and temporal lag between the two time series, phase evolution of each time series was computed from its wavelet transform at the 27–31 months periodic scale (see Methods and Appendix S1). Figure 4C demonstrates the resulting phase evolution of the homeless TB cases and their HIV+/PPD− contacts over the 13-year period in the data. The phase difference of the two time series was then computed by Equation (3) in Appendix S1 and is plotted in Figure 4D. The phase difference (in months) represents the lag time between the waves of the two time series at the 27–31 months periodic component where they appear to be highly correlated and synchronized. The mean lag time between the TB cases and their HIV+/PPD− contacts is 5.4 months for the entire 13-year period. As can be seen in Figure 3B, the four-year period from January 2002 to December 2005 is the longest period in the SFDPH TBCP data with the least amount of missing HIV-related information for the PPD− contacts. Specifically, there are only two PPD− contacts with unknown HIV status: one in November 2002 and one in July 2003. The mean lag time between the homeless TB cases and their HIV+/PPD− contacts for this four-year period, where HIV-related information is most complete, was estimated at 4.4 months with waves of TB infection following those of the HIV+/PPD−. Finally, to demonstrate the dichotomy in the dynamics of the two populations, we performed a similar analysis for the non-homeless TB cases and their HIV+/PPD− contacts. Figure 5 is the coherence plot of the non-homeless TB cases and their HIV+/PPD− contacts, where there is no evidence of consistently significant coherence at a periodic component between the two time series.

#### Missing data imputation: Verification of robustness

**Figure 6:**
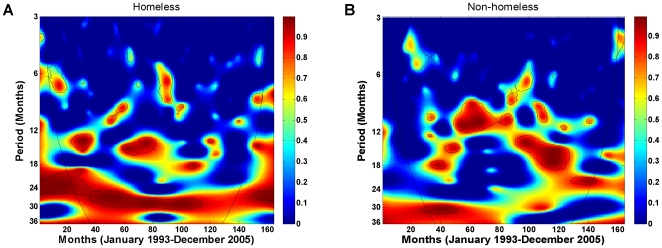
Robustness of coherence and synchrony between the TB cases and their HIV+/PPD− contacts, January 1993–December 2005. Wavelet coherence analysis was performed, where each PPD− contact with missing HIV status was randomly assigned a status (HIV+ or HIV−). The experiment was repeated 500 times and the average coherence was recorded. Each time series was log-transformed and standardized before wavelet analysis was performed. (A) Homeless: the two time series manifest high coherence at the same 27–31 months periodic band, as in Figure 4B, with the lag time of 5.2 months. (B) Non-homeless: similar to Figure 5, there is no evidence of a significant periodic component in spite of missing HIV data imputation.

To investigate the effect of missing HIV-related data on the statistical significance of association and synchrony between the TB cases and their HIV+/PPD− contacts, a randomized procedure was applied to the data as follows. Each PPD− contact with missing HIV status in the SFDPH TBCP data was randomly assigned a status (HIV+ or HIV−), and coherence analysis was performed. This experiment was repeated 1000 times and the average coherence was recorded. The result is demonstrated in Figure 6A, where it can be clearly seen that the key periodic component of Figure 4 is preserved. In spite of randomized missing data imputation, the two time series manifest high coherence at the 27–31 periodic scale with the average lag time of 5.2 months for the entire 13-year period, a finding quite similar to that of the original data with missing HIV data (Figure 4). To verify that the observed robustness of the results is unique to the homeless and not an outcome of chance, we applied the same randomization procedure to the TB surveillance data for the non-homeless. The result is illustrated in Figure 6B, where there is no evidence of coherence at any periodic scale over the entire period. While this may suggest that HIV infection is likely not a significant factor in sustaining the TB transmission among the San Francisco non-homeless, together these results with those of previous section suggest that the TB surveillance data, in spite of missing HIV-related information, is sufficiently representative of the underlying disease frequency among the homeless.

## Discussion

Using extensive TB surveillance data on the San Francisco homeless, we found that the evolution of TB cases and their HIV+/PPD− contacts is significantly interdependent, synchronized at about every 2.5 years and separated by the mean lag time of 5.4 months.

**Figure 7:**
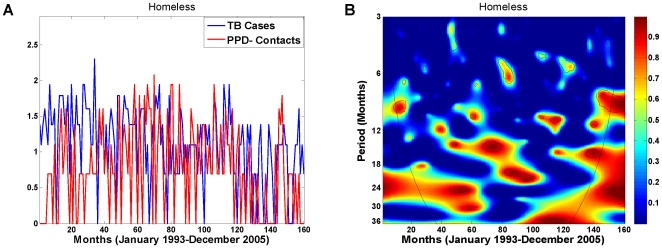
Wavelet time series analysis of the homeless TB cases and their PPD− contacts, January 1993–December 2005. (A) Log-transformed time series of the TB cases and their PPD− contacts. (B) Wavelet coherence between the homeless TB cases and their PPD− contacts. There is no evidence of a consistent periodic component and coherence between the two time series at any time scale (compare with Figure 4B).

What generates the observed association and synchrony in the two time series? The SFDPH TBCP begins active contact tracing soon after the detection of new TB cases, which can take a few months before all contact are traced and their risk factors are identified. Thus, it would seem reasonable to suggest that the co-evolution of TB cases and their HIV+/PPD− contacts is likely a confounding effect of contact tracing. If contact tracing is to explain the joint dynamics between the TB cases and their HIV+/PPD− contacts, then surely similar dynamics must exist between the TB cases and their PPD− contacts, albeit with a shorter lag time since it may take longer to identify the HIV status of all contacts. To investigate, we applied similar wavelet coherence and phase analyses to the data on the homeless TB cases and their PPD− contacts, the same subpopulation from which data on HIV+ individuals were extracted. Figure 7 illustrates both time series (Figure 7A) and the outcome of their coherence analysis (Figure 7B). As can be clearly seen, there is no evidence of stable coherence and synchrony over time between the TB cases and their PPD− contacts. Thus, contact tracing alone is not sufficient to rationalize the observed joint dynamics between the TB cases and their HIV+/PPD− contacts.

What other processes can potentially explain these trends? Although it is well established that HIV plays a key role in the sustenance and amplification of TB transmission in high-risk populations, the epidemiology of their interaction is not well understood [17]. Clearly, any significant epidemiological interaction must manifest itself, among other mathematical representations, through a high degree of coherence and synchrony at some periodic scale between TB and HIV within a population (e.g., homeless). Although we demonstrated significant coherence and synchrony between the homeless TB cases and their HIV+/PPD− contacts, comparable independent HIV data on the homeless population through HIV/AIDS surveillance are required to provide sound evidence of such an interaction. Thus, while we demonstrated that the codependency between the evolution of TB cases and their HIV+/PPD− contacts is significant, unique to the homeless, not an outcome of chance, and contact tracing cannot fully account for these trends, it remains an open problem as to what epidemiological mechanisms generate and govern the joint dynamics of HIV and TB among the homeless.

Thus, a limitation of this study is in the absence of independent data on homelessness, irrespective of HIV or TB status, and HIV-related data on the homeless population, irrespective of their TB status. Recall that the primary role of the SFDPH TBCP is to conduct active surveillance with respect to TB, and not HIV, in San Francisco. As a result, we sought to obtain HIV data independently collected on the homeless population through HIV/AIDS surveillance. However, the extent and completeness of the HIV-related data through TB surveillance were not matched by similar data obtained from direct HIV surveillance by the AIDS office in San Francisco. This is likely because active HIV/AIDS surveillance is mostly targeted at other high-risk populations for HIV transmission, such as men having sex with men, and not at the indigent populations with poor access to health care and equally or more severely affected by HIV and AIDS. Similarly, we were not able to obtain data on homelessness, including the size and time of influx of new individuals into the homeless population. Joint analyses of these data coupled with those from TB surveillance should greatly enhance understanding of the multi-scale epidemiological interaction between TB and HIV, leading to adoption of more effective control measures and intervention policies.

Two important findings of this study are worthy of note. First, while the prevalence, or seroprevalence, of HIV infection among the TB-infected homeless population in San Francisco remains unknown, using the SFDPH TBCP data, we demonstrated that it must be much higher than that of the general population of the United States, exclusive of California [18], that of the San Francisco general population [19], and even that of the San Francisco homeless [6], [7]. Second, we demonstrated that HIV status reporting has improved markedly among the San Francisco homeless compared to the non-homeless (Table 1 and Figures 2, 3, S1, S2), and the U.S. general population, exclusive of California [18]. The observed coherence and synchrony together with these two findings further substantiate the premise that HIV is likely a key factor in the sustenance of TB transmission among the San Francisco homeless.

Finally, the findings of this study have clear implications for public health policy. First, the voluntary nature of testing and reporting of HIV status presents a challenge to the surveillance and control of HIV and TB infection in high-risk populations. Our study highlights the importance of HIV-related data for investigating the association between HIV and TB infection in high-risk populations, and reconfirms the need for simplifying the current procedures for obtaining written consent for HIV testing [33]. Second, high prevalence of HIV infection among the San Francisco homeless, together with the observed association and synchrony between the TB cases and their HIV+/PPD− contacts, underscore the need to revisit the current reductionist approach to public health surveillance. Traditionally, public health departments are divided into distinct disease surveillance programs. However, separate surveillance programs cannot effectively address problems that arise due to interactions between multiple diseases in vulnerable populations. The strong interdependency in the epidemiology of TB cases and their HIV+ contacts among the San Francisco homeless, where the dynamics of the two were shown to be significantly intertwined, embodies this assertion. This substantiates the notion that, to effectively reduce the TB transmission among the San Francisco homeless, the TB surveillance efforts would have to be coupled with those of HIV.

## Supporting Information

Figure S1 Annual percentage of HIV+ TB cases, January 1993-December 2005. (A) Homeless. (B) Non-homeless. The slope of the linear fit (red line) indicates the rate of growth for the homeless (positive value) and the rate of decline for the non-homeless (negative value).
(0.06 MB DOC)

Figure S2 Annual percentage of PPD+ contacts with missing HIV status, January 1993-December 2005. (A) Homeless. (B) Non-homeless. The slope of the linear fit (red line) indicates the rate of decline for the homeless (negative value) and the rate of growth for the non-homeless (positive value).
(0.06 MB DOC)

Appendix S1 Wavelet Time Series Analysis
(0.07 MB DOC)
