# Supplementary figures and images for: Empirical Evidence for Synchrony in the Evolution of TB Cases and HIV+ Contacts among the San Francisco Homeless
Source: PLoS One. 2010 Jan 22;5(1):e8851. doi: 10.1371/journal.pone.0008851 (PMC2809753; doi:10.1371/journal.pone.0008851)

**FIGURE S1**

**A**


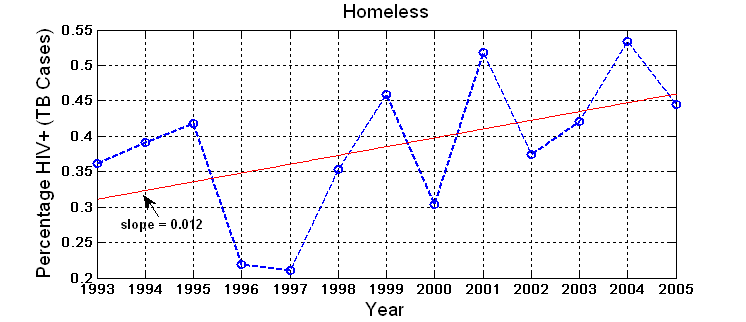


**B**

**
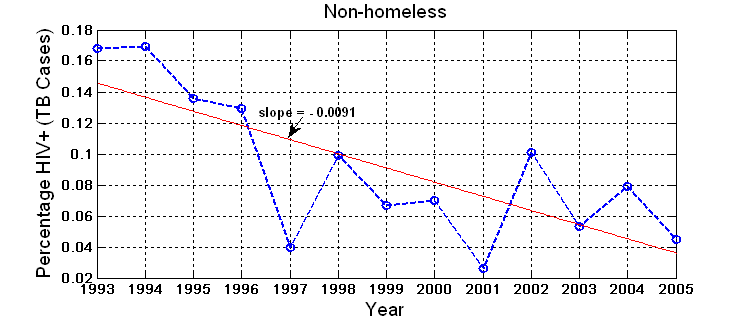
**

Supplement: Figure S1 — Annual percentage of HIV+ TB cases, January 1993-December 2005. (A) Homeless. (B) Non-homeless. The slope of the linear fit (red line) indicates the rate of growth for the homeless (positive value) and the rate of decline for the non-homeless (negative value). (0.06 MB DOC) [file pone.0008851.s001.doc]

**FIGURE S2**

**A**


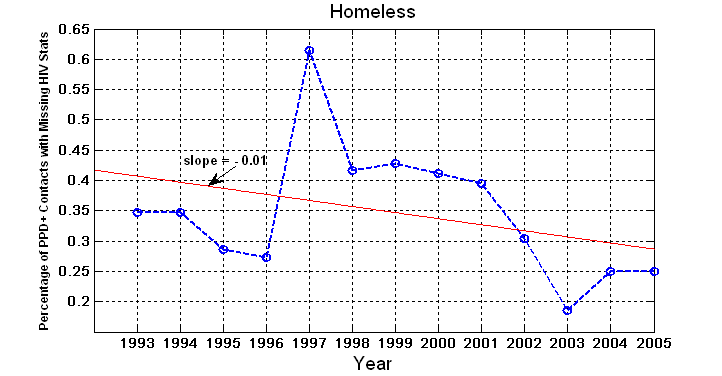


**B**

**
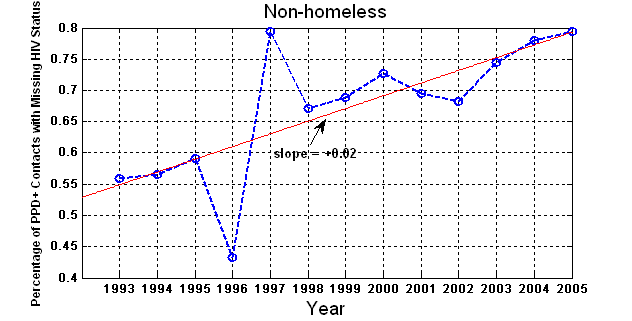
**

Supplement: Figure S2 — Annual percentage of PPD+ contacts with missing HIV status, January 1993-December 2005. (A) Homeless. (B) Non-homeless. The slope of the linear fit (red line) indicates the rate of decline for the homeless (negative value) and the rate of growth for the non-homeless (positive value). (0.06 MB DOC) [file pone.0008851.s002.doc]
