# Supplementary material for: Empirical Evidence for Synchrony in the Evolution of TB Cases and HIV+ Contacts among the San Francisco Homeless
Source: PLoS One. 2010 Jan 22;5(1):e8851. doi: 10.1371/journal.pone.0008851 (PMC2809753; doi:10.1371/journal.pone.0008851)
Supplement: Appendix S1 — Wavelet Time Series Analysis (0.07 MB DOC) [file pone.0008851.s003.doc]

**Appendix S1**

Wavelet Time Series Analysis

Wavelet transform achieves good localization in both time and frequency by decomposing the signal into high- and low-frequency components. This is accomplished by contracting or dilating the analysis window, which is a complex or real function (wavelet) (20-24). Each log transformed and standardized time series (see Methods) is wavelet transformed:

(1)

Here $s(t)$is the original time series, parameters *a* and $\tau$ represent the scale (or period) and time shift respectively, $\psi(t)$ is the wavelet chosen for the study, and $\psi^{*}(t)$ is the complex conjugate form of the wavelet. We chose the Morlet wavelet defined as for our study, which is a complex exponential. The local wavelet power spectrum of a signal is then where ‘|| ||’ denotes the magnitude of the wavelet coefficients evaluated at the scale *a*, and the time shift$\tau$. The statistical association or correlation between the spectra of two time series can be quantified using the wavelet coherence (24):

(2)

The numerator represents the cross-spectrum between two time series$s_{1},s_{2}$. Thus the wavelet coherence is the cross-spectrum normalized by the product of the local power spectrum of each time series. Because the Morlet wavelet is a complex function, and thus the resulting wavelet coefficientsare complex numbers, the phase of a wavelet transformed time series can be written as the inverse tangent of the ratio of the imaginary part over the real part of (25). Analogously, the phase difference between two time seriescan be interpreted as the ratio of the imaginary part of the cross-spectrum of the two time series,, over the real part of . Thus, the phase difference indicates the statistical tendency for the two signals to be phase locked:

(3)

In Equation (3), is the imaginary part, and is the real part of the cross-spectrum between the two series. At and near the beginning and end of a time series, there is tendency for loss in statistical power, a phenomenon referred to as the edge effects (26). The cone of influence, depicted as a parabola in Figures 4B, 5, 6, and 7, captures this effect, where the statistical power in the area outside the parabola should be interpreted with caution. Both time series were padded with zeros up to the next highest power of two to reduce the bias due to the edge effects.

To determine whether the observed association between the two time series is significant (alternate hypothesis) or random and due to chance (null hypothesis), its statistical significance must be assessed. In this study, we used bootstrap methods (27) similar to those used in (28, 29). For each time series, a control series was constructed by permuting the original series while maintaining the statistical properties of the series. Then each similarly constructed control series underwent wavelet analysis and corresponding values were recorded. This procedure was repeated 400 times. Finally, the results of the original series were compared against the 95^th^ quantile of the resulting distributions.
